# Unraveling the Equine Lymphocyte Proteome: Differential Septin 7 Expression Associates with Immune Cells in Equine Recurrent Uveitis

**DOI:** 10.1371/journal.pone.0091684

**Published:** 2014-03-10

**Authors:** Roxane L. Degroote, Stefanie M. Hauck, Barbara Amann, Sieglinde Hirmer, Marius Ueffing, Cornelia A. Deeg

**Affiliations:** 1 Institute of Animal Physiology, Department of Veterinary Sciences, Ludwig Maximilians University Munich, Munich, Germany; 2 Research Unit Protein Sciences, Helmholtz Center Munich, German Research Center for Environmental Health, Neuherberg, Germany; 3 Center for Ophthalmology, Institute for Ophthalmic Research, Eberhard Karls University of Tübingen, Tübingen, Germany; Oregon Health & Science University, United States of America

## Abstract

Equine recurrent uveitis is a spontaneous, lymphocyte-driven autoimmune disease. It affects horses worldwide and presents with painful remitting-relapsing inflammatory attacks of inner eye structures eventually leading to blindness. Since lymphocytes are the key players in equine recurrent uveitis, we were interested in potential changes of their protein repertoire which may be involved in disease pathogenesis. To create a reference for differential proteome analysis, we first unraveled the equine lymphocyte proteome by two-dimensional sodium dodecyl sulfate - polyacrylamide gel electrophoresis and subsequently identified 352 protein spots. Next, we compared lymphocytes from ERU cases and healthy horses with a two-dimensional fluorescence difference in gel electrophoresis approach. With this technique, we identified seven differentially expressed proteins between conditions. One of the significantly lower expressed candidates, septin 7, plays a role in regulation of cell shape, motility and migration. Further analyses revealed T cells as the main cell type with decreased septin 7 abundance in equine recurrent uveitis. These findings point to a possible pathogenetic role of septin 7 in this sight-threatening disease.

## Introduction

Equine recurrent uveitis (ERU) is a highly prevalent disease in horses and presents with spontaneously occurring, painful remitting-relapsing inflammation of inner eye structures [1]. Prior to an uveitic attack, activated peripheral-blood derived lymphocytes infiltrate the eye by crossing the blood-retinal barrier and destruct their main target, the retina [2]–[4]. With every subsequent relapsing inflammatory phase, lymphocyte infiltration from periphery reoccurs and inflammation increases in severity eventually leading to blindness [5]. Not only does this organ-specific autoimmune disease have severe, sometimes fatal consequences for diseased horses, it is also the only spontaneous model for relapsing autoimmune uveitis in man, due to remarkable clinical and immunopathological similarities [6]. Although it is known that in ERU, autoaggressive lymphocytes are predominantly targeted against retinal autoantigens [2], [7]–[9] and epitope spreading is a possible explanation for remitting-relapsing character of disease [10], underlying molecular mechanisms affecting lymphocyte function in ERU are still elusive. Changes in protein expression pattern of these immune cells might be a potential indicator for altered lymphocyte function contributing to pathogenesis. Since autoaggressive lymphocytes are present in peripheral blood directly before onset of an uveitic attack [10], differential proteome analysis of peripheral blood-derived lymphocytes in ERU is a valuable technique to gain further insights into these pathological processes. To create a solid basis for these analyses, however, knowledge of the equine lymphocyte protein repertoire is essential. Therefore, we unraveled the equine lymphocyte proteome and subsequently used two-dimensional fluorescence difference in gel electrophoresis (2D-DIGE) to screen the lymphocyte proteome for differences in protein abundance comparing peripheral lymphocytes of healthy horses and ERU cases. Taken together, this study aimed at finding differentially expressed proteins which might affect lymphocyte function and thereby contributing to pathogenesis of ERU.

## Materials and Methods

### Ethics statement

No experimental animals were used in this study. Horses were treated according to the ethical principles and guidelines for scientific experiments on animals according to the ARVO statement for the use of animals in Ophthalmic and Vision research. Blood from ERU horses was withdrawn as part of patient's diagnostics. Withdrawal of blood samples from healthy horses was permitted by the local authority (Regierung von Oberbayern; permit number: AZ 55.2-1-54-2532.3-21-12).

### Selection of animals used in the study

All ERU diseased horses were those brought to the Equine Clinic of the LMU Munich. ERU was diagnosed by clinical signs of acute uveitis accompanied by a documented history of recurrent eye inflammation. Horses with ERU included in this study had had at least three uveitic attacks. Blood from ERU cases was withdrawn prior to therapeutic pars plana vitrectomy, in quiescent stage of disease. The horses used in this study received topical medication solely to the eye, if at all, but did not receive systemical medication. Therefore, no influence was taken through treatment on the peripheral lymphocyte population investigated. Healthy horses used as controls were matched in sex and age.

### Sample preparation

Lymphocytes from 29 healthy horses and 27 ERU cases were examined in this study. In detail, peripheral blood derived lymphocytes (PBL) of 1 healthy horse were used for two-dimensional lymphocyte proteome reference map, 5 healthy controls and 5 ERU diseased horses were used for 2D-DIGE screening experiment. For Western blot analysis, lymphocytes of 12 healthy controls and 11 ERU cases were used. PBL from 11 healthy horses and 11 ERU cases were analyzed by flow cytometry. All blood samples from ERU diseased horses were obtained from the Equine Clinic in Munich without prior selection for a respective experimental condition (DIGE profiling, Western blot verification, flow cytometry). Diseased and healthy horses were matched in age and sex for DIGE screening experiments. Equine venous blood was collected in lithium-heparin coated tubes (Kabe, Nümbrecht-Elsenroth, Germany). After rough sedimentation of erythrocytes, lymphocytes were isolated from plasma by density gradient centrifugation (room temperature (RT), 290 relative centrifugal force (rcf), 25 min, brake off) using Biocoll separating solution (Biochrom, Berlin, Germany). Lymphocytes were extracted from intermediate phase, washed twice in PBS (4°C, 453 rcf, 10 min) and number of lymphocytes was counted to ensure comparable sample composition for further analyses. Cells were then either used immediately or stored at −20°C (pellet) or −80°C (vital cells, cryopreservated).

### Two-dimensional separation of the equine lymphocyte proteome

PBL of a healthy horse were dissolved in lysis buffer (9 M Urea, 2 M Thiourea, 1% Dithioerythritol, 4% CHAPS, 2.5 µM EDTA) and processed using QIAshredder homogenizers (Qiagen, Hilden, Germany) for depletion of DNA precipitates. Protein content of cell lysates was determined by Bradford protein assay (Sigma-Aldrich, Deisenhofen, Germany). One mg of lysate was loaded on 24 cm pH 3-11 NL IPG strips (GE Healthcare, Freiburg, Germany) by overnight reswelling and subjected to isoelectric focusing (IEF) on a 2117 Multiphor II Electrophoresis Unit with an Amersham Electrophoresis Power Supply EPS 3501 XL (Step 1: 2 h/50 V/2 mA/5 W, Step2: 11 h/600 V/10 mA/10 W, Step 3: 4 h/2000 V/10 mA/10 W, Step 4: 12 h/3000 V/10 mA/10 W). Strips were then equilibrated in 1% Dithiothreitol followed by 4.8% Iodoacetamide for 10 minutes each and sodium dodecyl sulfate - polyacrylamide gel electrophoresis (SDS-PAGE) was subsequently performed in an Ettan DALT Six Electrophoresis Unit 230 (GE Healthcare) with an Amersham Electrophoresis Power Supply EPS 3501 XL (Step 1: 45 min/600 V/150 mA/9 W, Step 2: 1 h/850 V/300 mA/60 W, Step 3: 8 h/1000 V/400 mA/90 W). Resulting gels were colloidal coomassie stained and scanned on a transmission scanner. As many spots as possible were cut from gels and processed for mass spectrometry.

### Two-dimensional fluorescence difference in gel electrophoresis (2D-DIGE)

Approximately 1×10^8^ PBL of 5 ERU diseased horses and 5 controls each were dissolved in DIGE lysis buffer (7 M Urea, 2 M Thiourea, 4% CHAPS, 30 mM Tris; pH 8.5) and processed using QIAshredder homogenizers for depletion of DNA precipitates (Qiagen). 50 µg protein of each sample was labeled separately with 400 pmol Cy3 or Cy5 fluorescent CyDyes (GE Healthcare) according to minimal labeling technique. To exclude possible dye-specific effects of labeling on one group, we applied reverse labeling technique, labeling 2 control PBL lysates with Cy3 and 3 controls with Cy5 and vice versa in ERU cases. Additionally, a pooled internal standard containing all 10 samples used in the experiment was labeled with Cy2 (GE Healthcare). Labeling of proteins at 4°C was terminated after 30 min by addition of lysine. After 10 minutes of incubation, samples were pooled into sets, each comprising three differently dyed 50 µg aliquots (diseased/control/internal standard). The 150 µg protein compounds were adjusted to a volume of 460 µl with lysis buffer, loaded on 24 cm pH 3–11 NL IPG strips (GE Healthcare) and subjected to IEF followed by equilibration and SDS-PAGE. Resulting gels were first scanned at different wavelengths (488 nm for Cy2, 532 nm for Cy3, 633 nm for Cy5 labeled proteins) with Typhoon Trio69 Scanner (GE Healthcare) and then silver stained for visualization of protein spots.

### Image analysis and detection of differentially expressed proteins (DeCyder software)

Data of scanned DIGE gels were imported into DeCyder 6.5 software (GE Healthcare) and processed in DIA module for separate analysis of each gel (intra gel analysis), comprising the assignment of dye tag to images (internal standard: Cy2, control: Cy3 or Cy5, diseased: Cy5 or Cy3 due to reverse labeling), spot detection and normalization of Cy3 and Cy5 labeled spot abundances to the internal standard as well as comparison of spot abundances between control and ERU data sets. To avoid detection of “false” spots, inclusion and exclusion criteria for spot detection were set at: spot slope >2, spot volume <30000, threshold 2.5. After automatic detection and matching of spots by software analysis was verified manually and corrected if necessary. In BVA module, standardized protein spot abundances from each gel data set generated in the experiment were compared (inter gel analysis), enabling detection of protein abundance differences between groups. Detected differences in protein abundance were considered significant at p<0.05 (Student's *t* test) and inclusion criterion for further analysis was a fold change of >1.5. Differentially abundant protein spots from digital DeCyder map were located on matching silver stained gels and excised for subsequent identification by mass spectrometry (MALDI/TOF-TOF; ABI 4700 Proteomics Analyzer, Applied Biosystems, Darmstadt, Germany).

### Identification of proteins from spotmap as well as differentially expressed proteins with mass spectrometry (MALDI-TOF/TOF and LC-MSMS)

Selected spots from 2D-gels were excised manually and silver stained spots were destained by repetitive washes in water and buffer containing 30 mM potassium ferricyanide and 100 mM sodium thiosulfate. Destained spots from silver stained gels as well as spots from coomassie stained gel were shrunk in 100% acetonitrile (ACN), rehydrated in 50 mM NH_4_HCO_3_ (shrinking and rehydration was performed twice) and dried in a SpeedVac centrifuge. Spots were then digested with 0.01 mg/ml trypsin (Sigma-Aldrich) in 50 mM NH_4_HCO_3_ overnight at 37°C. The supernatant was collected and combined with eluates of subsequent elution steps with 80% ACN and 0.1% trifluoroacetic acid (TFA). The combined eluates were dried in a SpeedVac centrifuge and dissolved in 50% ACN and 0.1% TFA.

For protein identification with MALDI-TOF/TOF mass spectrometry, 0.5 µl of a 1∶1 mixture of sample and a matrix solution consisting of 2.5 mg/ml a-cyano-4-hydroxy-cinnamic acid (Bruker, Bremen, Germany) were spotted on a MALDI target. Mass spectra were acquired using a Proteomics Analyzer 4700 mass spectrometer (Applied Biosystems). For each MS spectrum, 2500 laser shots were averaged and processed with external calibration. Peptide mass fingerprint (PMF) spectra were not smoothed and background was not subtracted. Monoisotopic peak masses were automatically determined within the mass range 800–4000 kDa with a signal to noise ratio minimum set to 5 and the local noise window width m/z 200. Up to eight of the most intense ion signals with signal to noise ratio above 30 were selected as precursors for MS/MS acquisition excluding common trypsin autolysis peaks and matrix ion signals. In MS/MS positive ion mode 4000 spectra were averaged with 1 kV collision energy, collision gas air at a pressure of 1.6×10-6 torr and default calibration. Monoisotopic peak masses were automatically determined with a signal to noise ratio minimum set to 10 and the local noise window width m/z 200. Combined PMF and MS/MS queries were performed using the MASCOT search engine (Matrix Science, London, UK; http://www.matrixscience.com) embedded into GPS-Explorer Software (Applied Biosystems).

LC–MSMS mass spectrometry was performed as previously described [11]. Briefly, the digested peptides were loaded automatically to a HPLC system (Thermo Fisher Scientific) equipped with a nano trap column in 95% buffer A (5% ACN, 0.1% formic acid (FA) in HPLC-grade water) and 5% buffer B (80% ACN, 0.1% FA in HPLC-grade water). After 5 min, the peptides were eluted and separated on the analytical column (75 µm inner diameter×15 cm, Acclaim PepMap100 C18, 3 µm, 100 Å, Dionex) by a gradient from 5% to 50% of buffer B at 300 nl/min flow over 120 min followed by a 5 min gradient from 50% to 100% buffer B in 5 min. The eluting peptides were analyzed online in a LTQ OrbitrapXL mass spectrometer (Thermo Fisher Scientific) coupled to the HPLC system with a nano spray ion source. The mass spectrometer was operated in the data-dependent mode to automatically switch between Orbitrap-MS and LTQ-MS/MS acquisition. Survey full scan MS spectra (from m/z 200 to 1500) were acquired in the Orbitrap with high-resolution (60,000 full-width half maximum). The method used allowed sequential isolation of the most intense ions (up to ten), depending on signal intensity, for fragmentation on the linear ion trap using collisional induced dissociation at a target value of 100,000 ions. High-resolution MS scans in the Orbitrap and MS/MS scans in the linear ion trap were performed in parallel. Target peptides already selected for MS MS/MS were dynamically excluded for 30 s.

MALDI PMF and MSMS spectra as well as LC-MSMS-derived MS/MS spectra were analyzed using Mascot (version 2.2, Matrix Science, London, UK; http://www.matrixscience.com), set up to search the Ensemble Horse protein database (version 2.66, 12722794 residues, 22644 sequences, http://www.ensembl.org) setting trypsin as digestion enzyme and allowing fragment ion mass tolerance of 0.3 Da and a parent ion tolerance of 65 ppm for MALDI analyses. One missed cleavage was allowed and iodacetamide derivatives of cysteines as stable modifications as well as oxidation of methionine and deamidation of asparagine and glutamine as variable modifications were specified for Mascot searches.

Protein identifications were accepted if the probability based MOWSE protein score was above the p<0.01 significance threshold for the database and contained at least two identified peptides with at least 80.0% probability as specified by the Peptide Prophet algorithm [12]. Additionally, theoretical isoelectric point (p*I*) and molecular weight (Mw) from most search results correlated to the position of the corresponding spot in the gel. Proteins that contained similar peptides but could not be differentiated based on MS/MS analysis alone were grouped to satisfy the principles of parsimony.

### Quantification of septin 7 expression differences (Western blot)

Lymphocytes were lysed in lysis buffer (9 M Urea, 2 M Thiourea, 1% Dithioerythritol, 4% CHAPS, 2.5 µM EDTA) and incubated with laemmli buffer (4% SDS, 20% glycerol, 10% 2-mercaptoethanol, 0.004% bromphenol blue, 0.125 M Tris; pH 6.8) for 5 minutes at 95°C and 750 rpm on a bench top shaker. From each sample, 10 µg protein was separated by SDS-PAGE on 8% gels and blotted semidry onto PVDF membranes (GE Healthcare). Unspecific binding was blocked in 1% Polyvinylpyrrolidone with 0.05% Tween20 (PVP-T) for 1 h at room temperature. Blots were incubated with mouse anti-septin 7 antibody (SantaCruz Biotechnology, Heidelberg, Germany, 1∶1000) at 4°C overnight. Before and after incubation with HRP-coupled anti-rabbit IgG antibody (Sigma-Aldrich, 1∶3000) for 1 h at room temperature, blots were washed in phosphate buffered saline solution with 0.05% Tween20 (PBS-T). Signals were detected by enhanced chemiluminescence on X-ray film (SUPER-2000G ortho, Fuji; Christiansen, Planegg, Germany). Films were scanned on a transmission scanner and densitometric quantification of Western blot signals was performed using ImageQuantTL software (GE Healthcare). Specific binding of septin 7 antibody to horse septin 7 was verified with immunoprecipitation and subsequent mass spectrometry analysis, clearly identifying equine septin 7 bound by the antibody. Blots were then incubated in stripping buffer (100 mM NaOH, 2% SDS, 0.5% DTT) for 1 hour at 55°C, washed with PBS-T and blocked with 1% PVP-T for 1 hour at room temperature. After repeated washing with PBS-T, blots were re-incubated with mouse anti-beta actin antibody (Sigma-Aldrich; 1∶5000) followed by secondary HRP-coupled anti-mouse IgG antibody (Sigma-Aldrich, 1∶5000) and signals were developed as described above. Subsequently, all septin 7 signal abundances were normalized to respective beta actin values. Statistical analysis of septin 7 expression level comparison between ERU samples and controls was performed using Student's *t* test and differences in protein expression were considered significant at p<0.05.

### Analysis of septin 7 expression differences with flow cytometry

Equine PBL were isolated from plasma by density gradient centrifugation as described above. Cell staining was performed either directly (CD4, CD8, CD21) or after permeabilization of cells (septin 7) in 96 well roundbottom plates with 1×10^6^ cells per well. Mouse IgG1 anti-equine CD4, mouse IgG1 anti-human CD21 (both Serotec, Puchheim, Germany, 1∶10) and mouse IgG3 anti-equine CD8α (VMRD, Pullman, Washington, United States, 1∶50) antibodies were diluted in staining buffer (1% BSA + 0.001% NaN3 in PBS) and incubated with cells for 30 minutes at 4°C. After washing with staining buffer, respective secondary antibodies were added (anti mouse-IgG1:PE, SantaCruz, 1∶200 or anti mouse-IgG3:PE, Biozol, Eching, Germany, 1∶200) and incubated for 30 min at 4°C. Subsequently, cells were permeabilized (BD Cytofix/Cytoperm fixation/permeabilization kit; BD Biosciences, Heidelberg, Germany) and washed twice with staining buffer. Diluted anti-septin 7 antibody (SantaCruz, 1∶50) incubated with cells for 30 minutes at 4°C. After washing with staining buffer, anti-rabbit IgG:Alexa488 antibody (Invitrogen, Karlsruhe, Germany, 1∶200) was added for 30 minutes at 4°C. Cells were stored at 4°C in staining buffer with 1% PFA until further processing. Measurement of cells was performed on FACS Canto II with FACS Diva 6.1.3 software (both BD Biosciences). Lymphocytes were gated according to forward scatter (cell size) and side scatter (intercellular granularity) properties of cells. Compensation was performed manually. 5000 cells were measured per staining. Further analysis of flow cytometry data was performed using open source Flowing Software 2.5.0 (Perttu Terho, Turku Centre for Biotechnology, Finland).

## Results

### Two-dimensional separation of the equine lymphocyte proteome

To analyze the equine lymphocyte protein repertoire, we separated lymphocytes from healthy horses by 2D-PAGE. High-resolution protein pattern was made visible by colloidal coomassie staining (figure S1, representative 2D-gel with lymphocyte proteome pattern of one healthy horse; a total of 94 2D-PAGE experiments with different specimen were performed in this study) and showed single, well-separated protein spots with minimal streaking. 352 protein spots were unambiguously identified by mass spectrometry and represented 229 different proteins (figure S1 and table S1).

### Seven proteins show altered expression in ERU

**Figure 1:**
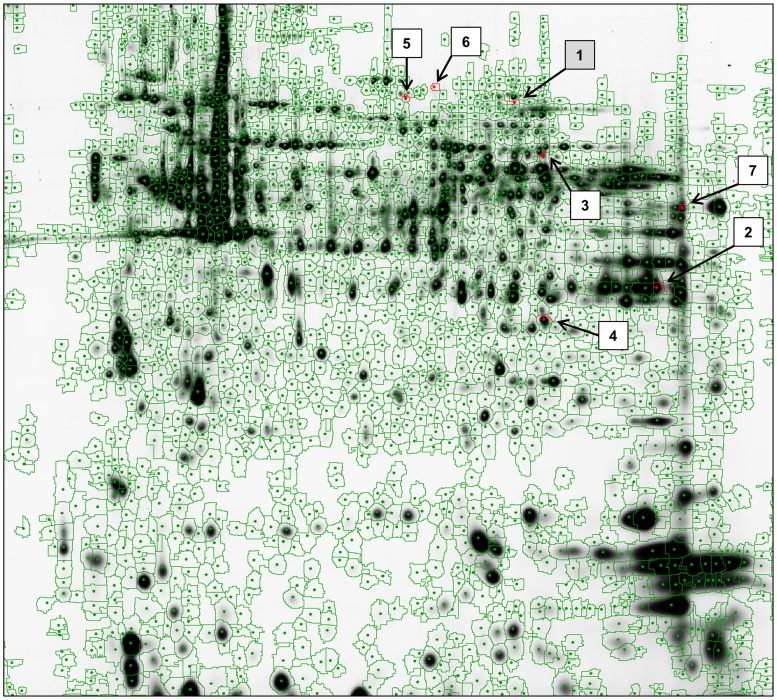
Spot map of equine PBL proteome generated by DeCyder 6.5 software. Protein spots detected on gel after scanning and processing were encircled in green. Spots with differential abundance between control- and ERU specimen (n = 5 each) were encircled in red and numbered according to table 1. Spot numbers referring to proteins with higher abundance in ERU were highlighted in grey, those with lower abundance in ERU are shown in white. Septin 7 spot (No. 7) showed diminished expression in ERU PBL proteome.

As we were interested in the characterization of lymphocyte protein expression pattern differences between healthy horses and ERU cases, we used 2D-DIGE technique for comparative proteome screening. With this approach, we were able to detect seven differentially abundant proteins in ERU (Figure 1, figure S2; p<0.05, fold change >1.5), which could clearly be identified by mass spectrometry (Table 1). One of these proteins, lactotransferrin, showed higher expression levels in lymphocytes of ERU cases, whereas six of the identified candidates were lower abundant in ERU. Among the latter were glyceraldehyde-3-phosphate dehydrogenase, protein tyrosine phosphatase non-receptor type6, voltage-dependent anion-selective channel protein 2, programmed cell death 6-interacting protein, ezrin and septin 7 (Table 1).

**Table 1:** Differentially expressed Proteins in ERU detected by 2D-DIGE and identified by mass spectrometry.

^a^No	^b^Protein Name	^c^Acc No	^d^Mw (kDa)	^e^p*I*	^f^Prot Score	^g^Pept Count	^h^Appear-ance	^i^Fold Change	^k^ *t* test	^l^Expr in ERU
1	Lactotransferrin	O77811	77	8.03	150	21	6 (15)	2.0	0.040	▴
2	Glyceraldehyde-3-phosphate dehydrogenase	P00355	36	8.32	170	10	15 (15)	1.6	0.017	▾
3	Tyrosine-protein phosphatase, non-receptor type 6	Q53XS4	68	8.08	92	15	6 (15)	2.1	0.008	▾
4	Voltage-dependent anion-selective channel protein 2	P68003	32	7.59	176	14	15 (15)	2.8	0.038	▾
5	Programmed cell death 6-interacting protein	Q8WUM4	96	6.33	69	12	12 (15)	2.2	0.027	▾
6	Ezrin	P15311	69	6.34	173	19	6 (15)	2.0	0.038	▾
7	Septin 7	Q6Q137	48	8.96	110	13	15 (15)	1.9	0.031	▾

Differentially expressed proteins in lymphocytes of spontaneous ERU cases. Spots were excised from silver stained 2D-DIGE gels and identified by MALDI-TOF/TOF mass spectrometry; proteins listed were identified with a probability score that was significant with p<0.05. (a) protein number as shown in figure 1, (b) protein name and (c) accession number as listed in Ensembl horse protein database (www.ensembl.org), (d) theoretical molecular weight and (e) theoretical isoelectric point of respective protein, (f) probability based MOWSE score; score is -10*Log(P), where P is the probability that the observed match is a random event. Protein scores greater than 60 were significant (p<0.05), (g) peptide count from MALDI-TOF/TOF analysis. Differential protein abundance was detected by DeCyder 6.5 software, providing (h) the appearance of each protein spot among the DeCyder map images of the experiment (number in parentheses: three images were generated per gel: control, ERU and internal standard, adding up to a maximum of 15 possible spot maps), (i) fold change (>1.5) and (k) the p-value for differential expression of proteins comparing healthy state and ERU cases (Student's *t* test). (l) Expression in ERU specimen was compared to controls.

### Septin 7 expression in lymphocytes of ERU cases decreases to 62% of physiological expression level

**Figure 2:**
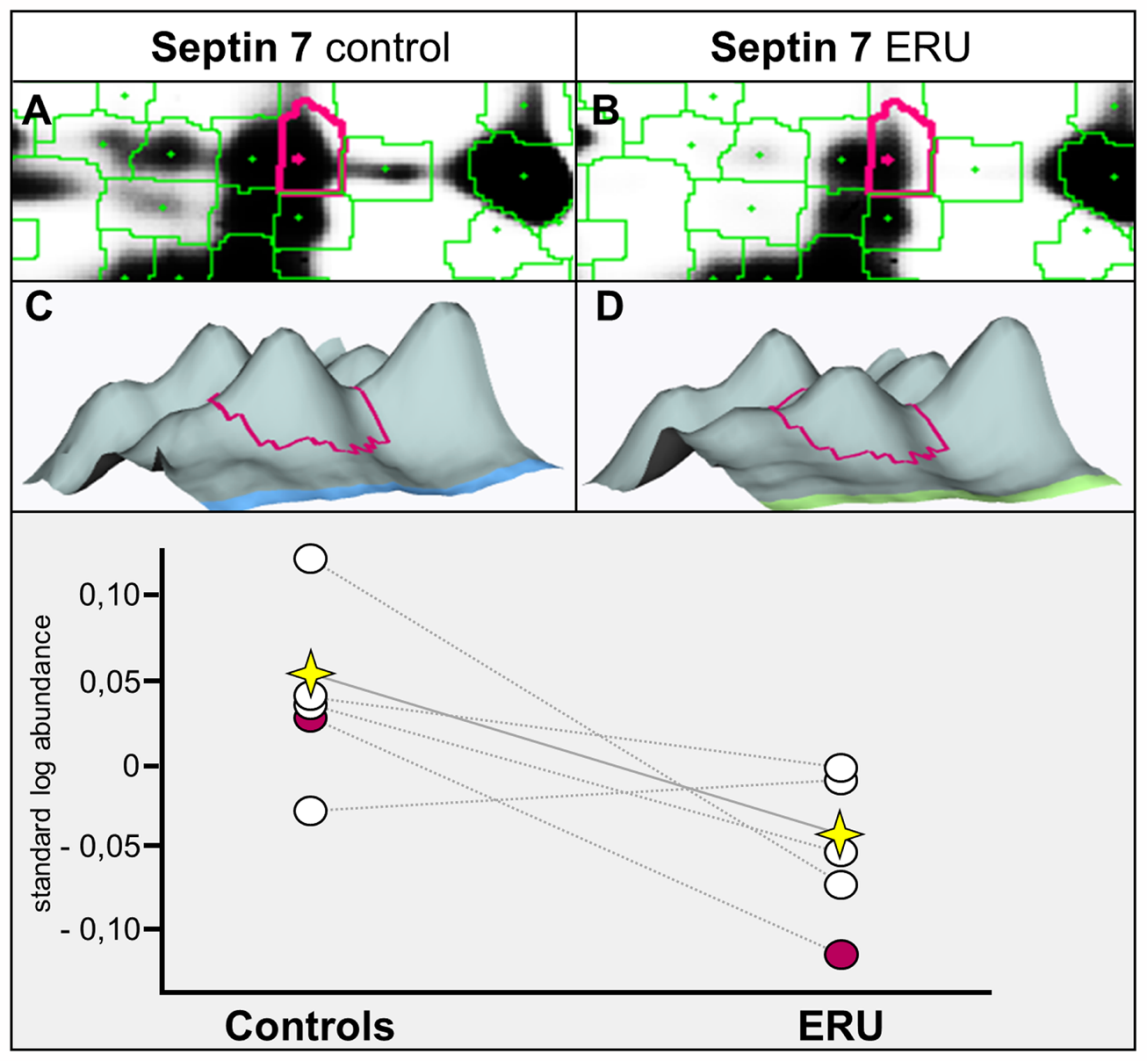
Septin 7 expression as detected in 2D-DIGE experiment. (A, B) Enlarged view of septin 7 spot from DeCyder-generated spot map. (C, D) Three-dimensional view of septin 7 spot. (Graph) Comparison of spot abundance on different gels (number of gels: 5; number of specimen: 10; proteins per gel were paired); spot highlighted in A–D is displayed in red, internal standard is displayed in yellow. Septin 7 clearly decreases in diseased state.

**Figure 3:**
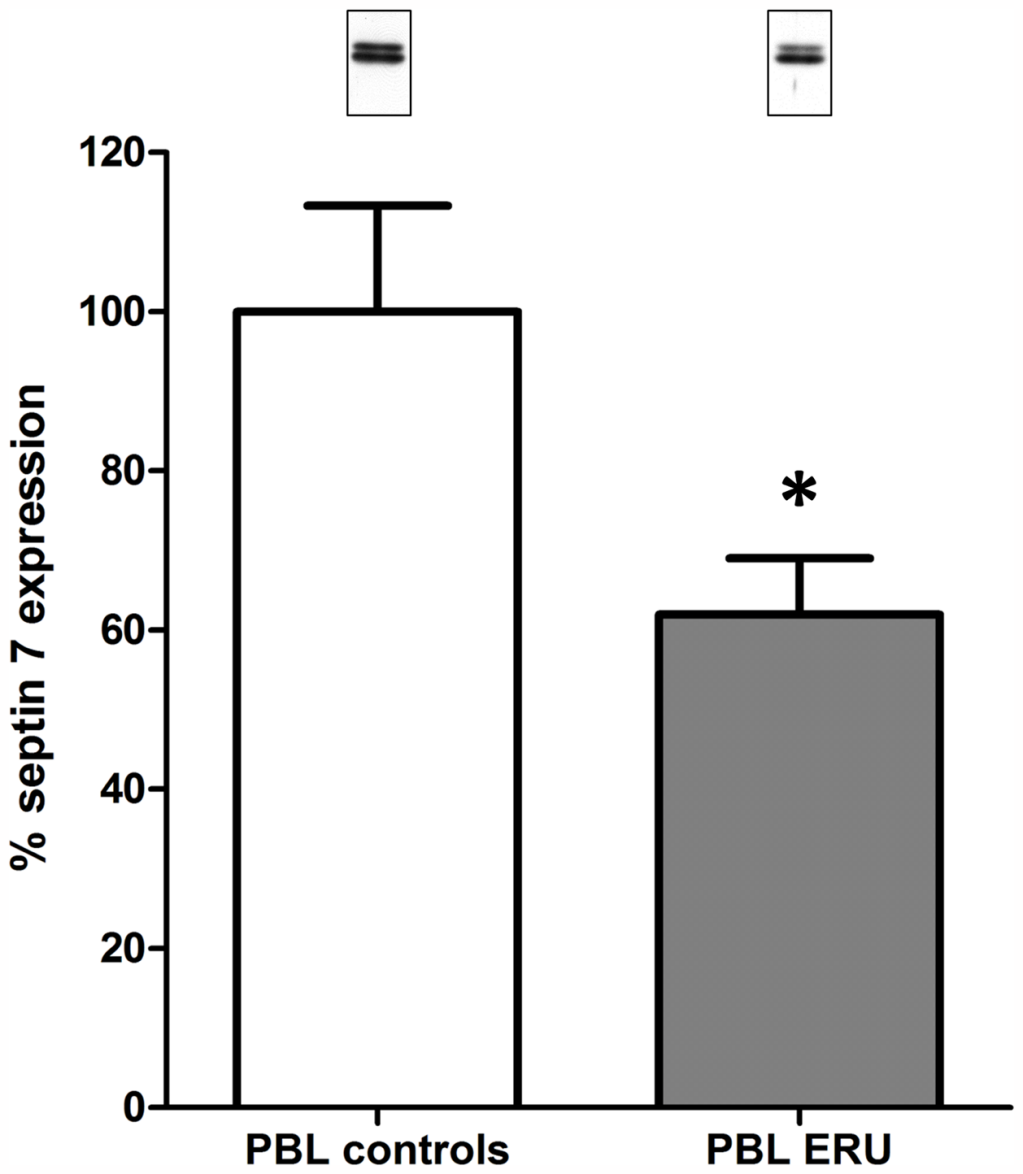
Septin 7 expression differences quantified and verified by Western blot. Septin 7 expression decreases in PBL of ERU diseased horses (n = 11, grey column, Septin 7 expression reduced to 62%) compared to PBL of healthy controls (n = 12, white column, set to 100%). Signal intensities of septin 7 were normalized to beta-actin abundances obtained after stripping and re-incubation of respective blots. Statistical analysis was performed using Student's *t* test (* p<0.05). Representative protein signals are shown above respective columns; upper Septin 7 signal was used for quantification, lower signal derived from unspecific binding of the antibody to beta actin and was not included in the analyses.

We decided to further analyze septin 7 (Figure 1, spot no. 7) due to its role in regulation of cell shape, motility and migration [13]. Spot analysis with DeCyder 6.5 software confirmed statistically significant differences in spot abundance (Student's *t* test p<0.05) (Figure 2 A–D) with a fold change of 1.9 between conditions (Table 1, column i). Furthermore, differentially abundant septin 7 spot was detected in every single gel included in analysis (Figure 2, Graph; Table 1 column h). Subsequently, expression differences of septin 7 between conditions found with 2D-DIGE technique was verified with Western blots, quantifying septin 7 abundance in a cohort of healthy horses (Figure 3, white column, average expression level set to 100%) in comparison to ERU cases (Figure 3, grey column). Average expression of septin 7 in ERU significantly decreased to 62% of expression level in controls, as determined by statistical analysis (Student's *t* test, p<0.05).

### Differential septin 7 expression is located in T cells

**Figure 4:**
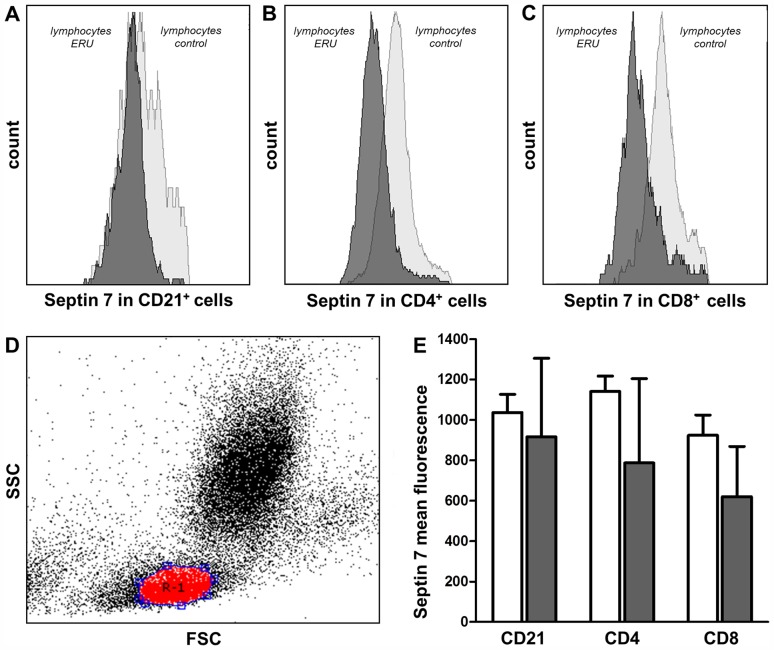
Characterization of septin 7 expression intensity on lymphocyte subsets by flow cytometry. Mean intensity of septin 7 expression decreases in lymphocyte subsets of ERU cases (n = 11, dark grey curve) compared to controls (n = 11, light grey curve). Histograms of representative specimen showed unchanged expression in B cells (A + E, CD21). In T cells, septin 7 expression intensity decreased to 75% (B + E, CD4) and 73% (C + E, CD8) of physiological expression level. Lymphocytes were gated according to forward- and sideward-scatter (D). Respective values of all 11 healthy and 11 ERU specimen used in this study are shown in graph (E).

Since we were interested in expression differences of septin 7 in lymphocyte subsets, we used flow cytometry for further analysis. To determine whether septin 7 expression differences were localized in B cells or T cells, we examined septin 7 expression in double staining with two T cell markers (CD4 and CD8) and one B cell marker (CD21) in lymphocyte populations of controls and ERU cases (Figure 4). Septin 7 was highly expressed in all lymphocyte subsets of controls (Figure 4 E). In B cells, septin 7 showed no change in expression intensity between conditions (CD21^+^ B cells; figure 4 A). In T cells, however, we found a clear decrease of septin 7 expression in ERU down to 75% (CD4^+^ T cells; figure 4 B) and 73% (CD8^+^ T cells; figure 4 C) of expression level in healthy specimen. Differences in septin 7 expression on lymphocyte subsets detected by flow cytometry were not statistically significant.

## Discussion

Equine recurrent uveitis is a lymphocyte-driven organ-specific autoimmune disease with high prevalence in horses [4], [14]. Starting spontaneously, painful inflammatory attacks of the inner eye alternate with quiescent phases [1]. Through the remitting-relapsing character of the disease, uveitic attacks become more and more severe over time and inner eye structures, predominantly the retina, are destroyed [3], [5], [7]. Up to date, therapy remains symptomatic, providing only moderate relief [15], [16]. Horses in advanced stage of disease often face enucleation of respective eye. If this involves both eyes, diseased horses have to be killed. Not only does ERU affect the well-being of the equine population worldwide, it also serves as the only spontaneous model for relapsing autoimmune uveitis in man [6] due to striking immunopathological and clinical resemblance such as comparable immune reactions to retinal S-antigen [2], [17]–[19], IRBP [7] and CRALBP [8], [9] as well as spontaneous onset of disease with remitting-relapsing character and unsolved etiology.

Prior to an uveitic attack, autoaggressive lymphocytes infiltrate the eye by crossing the BRB [20]. These cells can be isolated from peripheral blood of ERU cases and are a valuable biological source for analysis of disease-specific alterations in lymphocytes. To enable detection of possible protein expression differences in diseased state, we first needed detailed knowledge about the physiological protein repertoire of equine lymphocytes. Two-dimensional gel-based studies on physiological lymphocyte proteome were performed in several species [21]–[25], however, the horse was not analyzed so far. Using 2D SDS PAGE, we separated, displayed (figure S1) and subsequently identified 352 protein spots from the equine lymphocyte proteome (table S1), giving us detailed information about the equine lymphocyte protein repertoire and therefore a solid basis for further differential proteome analyses in ERU.

Previous gel-based differential proteome studies on lymphocytes in autoimmune disease by other research groups, such as rheumatoid arthritis in man and a mouse model for type 1 diabetes, led to the identification of several differentially expressed proteins speculated to be involved in disease pathogenesis [26], [27]. Apart from previous studies in our group concerning granulocytes in ERU [28], [29], however, to our knowledge, there were no gel-based studies on immune cells in autoimmune uveitis. Hence, with a comparative 2D-DIGE based screening of the equine lymphocyte proteome (Figure 1), we now aimed at finding differences in protein expression which may act as possible indicators of pathological abnormalities in lymphocytes of ERU cases. We decided to use biological, rather than technical replicates for a screening-study of a disease that occurs spontaneously among a heterogeneous group of individuals. Hence, we compared five biological replicates with outbred genetical background from healthy horses to five replicates from ERU diseased state and detected seven differentially expressed candidate proteins (Table 1). Since the individual variation in biological replicates masks the statistical significance of differential protein expression to a certain extent, proteins showing differential expression subsequent to this pre-selection might be especially robust in disease pathogenesis. However, biomarker candidates for the individual animal are possibly overlooked using this approach, letting personalized medicine to the side. Nonetheless, this screening-study resulted in the identification of a solid amount of differentially expressed proteins, none of which have been described in association with spontaneous autoimmune uveitis.

The enzyme glyceraldehyde-3-phosphate dehydrogenase [30] (figure S1 spot 263 and table 1) was not associated with autoaggressive lymphocytes so far. Further, this study describes the first identification of programmed cell death 6-interacting protein (figure S1 spot 44 and table 1), which may play a role in the regulation of both apoptosis and cell proliferation [31], as a downregulated lymphocyte protein in autoimmune disease.

Lactotransferrin (figure S1 spot 13 and table 1) acts as a growth stimulating factor for lymphocytes [32], [33] with favorable effects on the maturation and differentiation of T cells [34]. This is interesting regarding the fact that, in present study, higher expression of lactotransferrin in ERU points to the presence of activated immune cells. On the contrary, tyrosine-protein phosphatase non-receptor type 6 (figure S1 spot 123 and table 1), involved in signal transduction cascade of immune cells [35], was decreased in lymphocytes of ERU cases. Interestingly, this protein also showed decreased expression in T cells from patients with psoriasis, which led to enhanced inflammatory processes and autoimmune responses [36], therefore its role in immune cells is an interesting feature regarding pathogenesis of ERU as well.

Voltage-dependent anion-selective channel protein 2 (figure S1 spot 253 and table 1) is crucial for mitochondrial metabolism [37], [38]. Interestingly, this protein was described as a new candidate autoantigen for autoimmune uveitis in man [39]. Ezrin (figure S1 spot 79 and table 1) belongs to the protein family of cross linkers between the plasma membrane and the cortical cytoskeleton [40]. Ezrin's effect on enhanced T cell activation through changed expression has already been described in studies on rheumatoid arthritis [26] and humoral immunity [41]. The impact of its expression differences on disease pathogenesis in ERU needs to be further investigated in future studies.

In this study, we focused on diminished expression of Septin 7 (Figure 1 spot 7, figure 2, table 1, figure S1 spot 144, figure S2), due to its involvement in regulation of cell shape and motility [13], [42]. Septin 7 belongs to the evolutionarily conserved group of GTP-binding and filament-forming proteins originally discovered in yeast [43], [44] and recently described in vertebrates as important part of the cell division cycle [45]. Not only do septins interact with the actin cytoskeleton [46] and regulate microtubule stability within the cell [47], they also seem to coordinate changes in membrane organization of cells [42]. Septin 7 has a corset-like function providing cell compression and rigidity [13], [48], an interesting feature regarding pathogenesis of autoimmune disease, where activated immune cells cross anatomical barriers. Septin 7 was not described in association with autoimmune diseases to date, however, septin 7 was studied in other diseases such as acute myeloid leukemia, where septin 6, forming a complex with septins 2 and 7, showed significantly lower expression in the spinal cord of patients, indicating deficient regulation of cell cycle [49]. In neurodegenerative disease such as down syndrome, the diminished expression of septin 7 in diseased brain of human fetuses was connected to inhibition of synaptogenesis and synaptic function [50]. In neoplasia, especially in the brain, septin 7 was reported to be involved in malignant glioma cell growth due to its inhibitory effect on cell proliferation [51], which may also be of importance in ERU, where cells with diminished septin 7 expression could be those proliferating.

In blood-derived lymphocytes from ERU cases, septin 7 expression level decreased to 62% of expression level in controls (Figures 1+2, table 1; figure 3). Interestingly, further analyses of septin 7 expression on lymphocytes revealed that T cells are the predominant cell type with decreased septin 7 abundance in ERU. We found that septin 7 expression was diminished in both CD4+ and CD8+ lymphocytes (Figure 4 B + C), whereas B cells showed no expression difference between conditions (Figure 4 A). This finding is very interesting regarding the fact that, among all possible immune cells involved in autoimmune uveitis, T cells have the strongest impact on target tissue [11], [52]–[54]. The differential expression of septin 7 in these lymphocytes indicates association with mechanisms in disease pathogenesis. Septin 7 was recently reported to affect glucose uptake in cells [55]. In this study, 81% knock-down of septin 7 in podocytes increased their uptake capacity of glucose by affecting GLUT4 storage vesicle trafficking [55]. When lymphocytes shift from quiescent to activated state, their glucose metabolism increases drastically [56], [57]. Hence, decreased expression of septin 7 in T cells of ERU cases might additionally correlate with activated state of T cells in disease. This is supported by the fact that septin 7 is a potent inhibitor of cell proliferation [51] and its decrease in expression level may support inflammatory events in pathogenesis. Interestingly, Tooley et. al. earlier described altered migration characteristics in septin 7 deficient cells [13]. In respective study, a murine T cell line that expressed a short hairpin RNA to septin 7 was used, resulting in over 80% knock-down of septin 7 expression within the cell. Although this had an impact on coordinated crawling, septin 7 depletion allowed enhanced T cell migration through pores normally too narrow for passage of cells [13]. Hence, decreased septin 7 expression in equine lymphocytes of ERU cases might indicate changes in cytoskeleton and cell rigidity possibly leading to changes in migration ability. However, further studies are necessary to analyze possible connection of altered septin 7 expression and transmigration of cells in autoimmune uveitis.

The DIGE technique used as a discovery method in this study proved to be an effective tool for the detection of changes in the equine lymphocyte proteome of ERU cases. This was already shown in previous studies, where we detected differentially expressed talin 1 in the equine granulocyte proteome in ERU and subsequently identified its interactors [28], [29]. However, this method deprived us of the analysis of membrane-associated proteins since these tend to precipitate during IEF and are poorly soluble in aqueous solutions as those used in 2D-PAGE [58], [59]. Gel-free, detergent-based methods are more suitable for the analysis of this interesting fraction of lymphocyte proteins [11], [60] and will be performed in future studies to complete the analysis of proteome changes on inflammatory cells in ERU.

The detection of septin 7 downregulation in lymphocytes of cases with spontaneous autoimmune uveitis is a very interesting finding in our opinion and its functional pathogenetic role in ERU merits further analysis. Especially the connection of differences in septin 7 expression and the activation of autoreactive T cells deserves detailed studies, not only in peripheral blood-derived lymphocytes but also in intraocular cells, which accumulate in the vitreous in course of this sight-threatening disease.

## Supporting Information

Figure S1 **Representative map of the equine lymphocyte proteome.** Equine lymphocyte proteome of a healthy horse was separated by 2D-PAGE on 12% SDS gel loaded with 500 µg lymphocyte protein lysate, stained with colloidal coomassie (number of total lymphocyte 2D-PAGE experiments performed: 94). 352 protein spots were identified by mass spectrometry. Numbers of encircled spots correspond to protein identifications given in table S1.
(TIF)

Figure S2 **Differentially expressed proteins of the equine lymphocyte proteome as detected by DeCyder 6.5 software.** (A, B) Enlarged view of protein spot from DeCyder-generated spot map. (C, D) Three-dimensional view of protein spot. (Graph) Comparison of spot abundance on different gels (number of gels: 5; number of specimen: 10); every dot in graph represents respective protein in a different gel and condition (proteins per gel were paired), spot highlighted in A-D is displayed in red, internal standard is displayed in yellow.
(TIF)

Table S1 **Protein identifications from equine lymphocytes by mass spectrometry.** 352 protein spots (representing 229 different proteins) from equine lymphocytes identified by mass spectrometry; (column A) number of corresponding spot from master-gel in figure S1, (column B) protein name and (column C) accession number as listed in Uniprot protein database (www.uniprot.org), (column D) probability based MOWSE protein score from MALDI-TOF/TOF analysis; score is -10*Log(P), where P is the probability that the observed match is a random event. Protein identifications were accepted if the MOWSE score was above the p<0.01 significance threshold for the database and contained at least two identified peptides with at least 80.0% probability as specified by the Peptide Prophet algorithm. Percentage scores derived from identification of proteins with LC-MSMS mass spectrometry describe the percent probability of correct protein identification as well as the number of peptides sequenced (shown in parentheses), (column E) Peptide count from MALDI-TOF/TOF analysis. In comparison to a human lymphocyte proteome study by Vergara et al., where a total of 246 spots were identified, representing 174 different proteins [21], we could overall identify more proteins, however, the general protein pattern and identifications resemble each other in both species. Several of the differentially expressed proteins detected in the DIGE screening experiment (Figure 1 and table 1) were also present in the human proteome map. Septin 7 was not detected in the human lymphocyte proteome in that experiment, however, other septins were identified [21].
(XLSX)
