# Supplementary figures and images for: Unraveling the Equine Lymphocyte Proteome: Differential Septin 7 Expression Associates with Immune Cells in Equine Recurrent Uveitis
Source: PLoS One. 2014 Mar 10;9(3):e91684. doi: 10.1371/journal.pone.0091684 (PMC3951111; doi:10.1371/journal.pone.0091684)

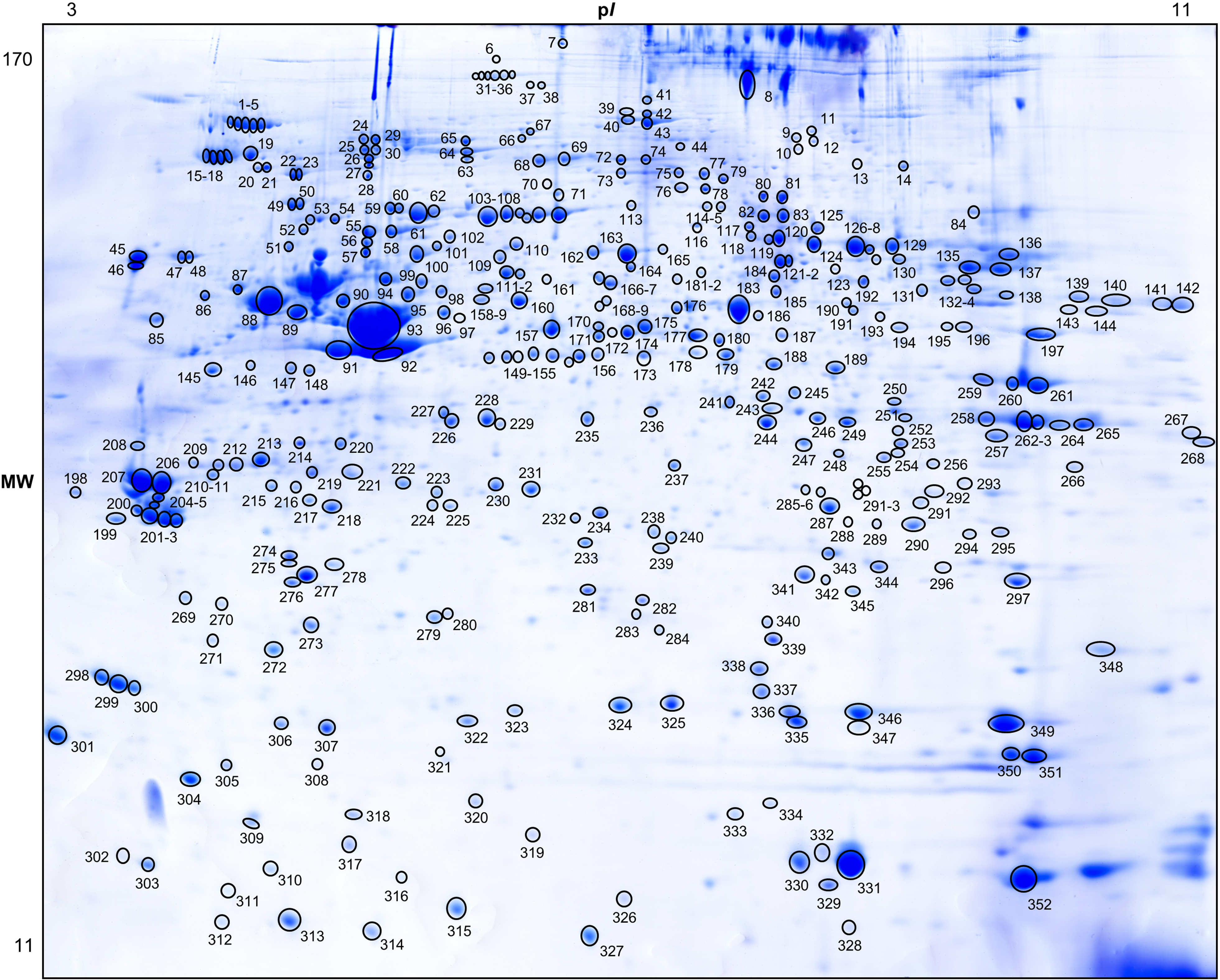

Supplement: Figure S1 — Representative map of the equine lymphocyte proteome. Equine lymphocyte proteome of a healthy horse was separated by 2D-PAGE on 12% SDS gel loaded with 500 µg lymphocyte protein lysate, stained with colloidal coomassie (number of total lymphocyte 2D-PAGE experiments performed: 94). 352 protein spots were identified by mass spectrometry. Numbers of encircled spots correspond to protein identifications given in table S1. (TIF) [file pone.0091684.s001.tif]

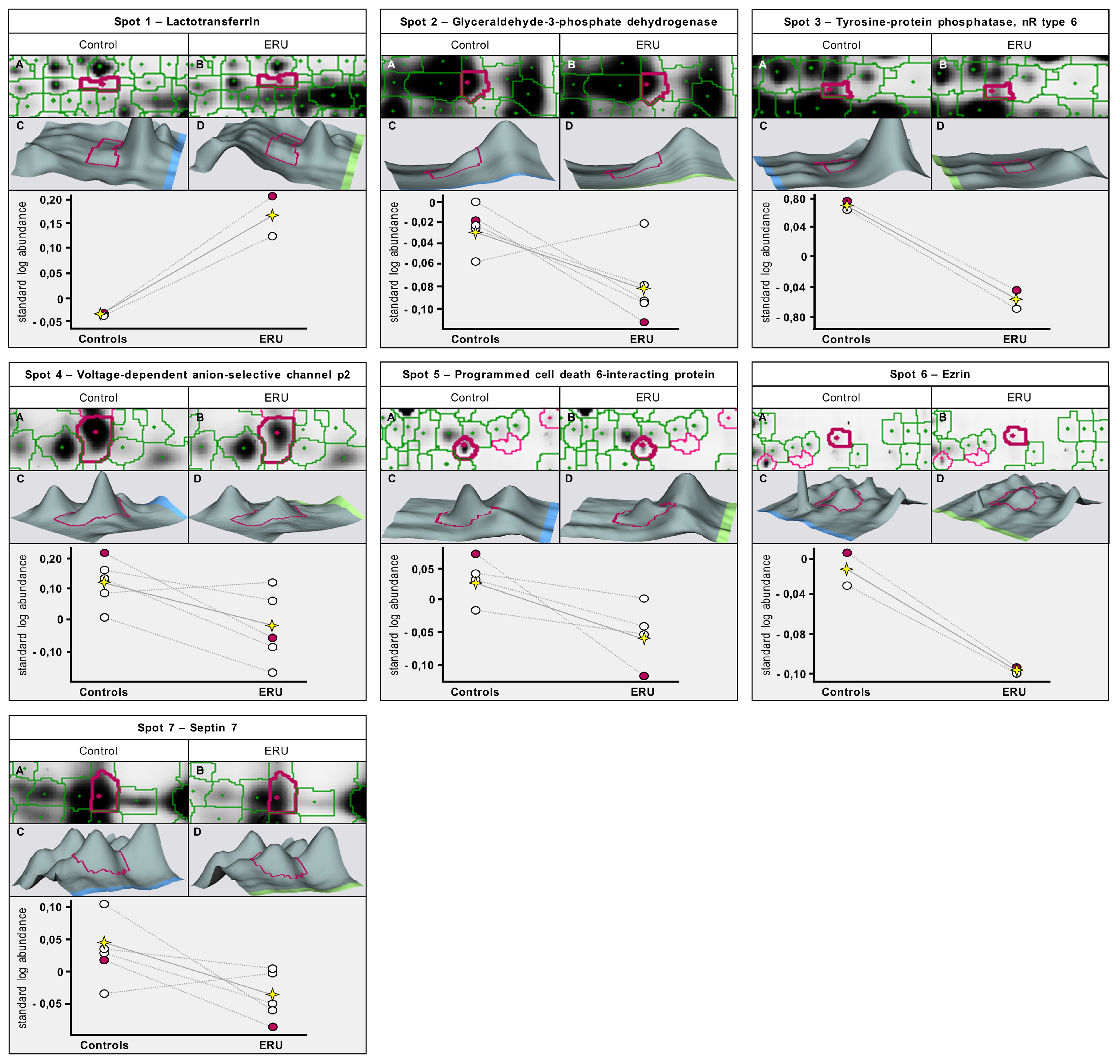

Supplement: Figure S2 — Differentially expressed proteins of the equine lymphocyte proteome as detected by DeCyder 6.5 software. (A, B) Enlarged view of protein spot from DeCyder-generated spot map. (C, D) Three-dimensional view of protein spot. (Graph) Comparison of spot abundance on different gels (number of gels: 5; number of specimen: 10); every dot in graph represents respective protein in a different gel and condition (proteins per gel were paired), spot highlighted in A-D is displayed in red, internal standard is displayed in yellow. (TIF) [file pone.0091684.s002.tif]
